# Supplementary material for: The Development of a Rabies Virus-Vectored Vaccine against Borrelia burgdorferi, Targeting BBI39
Source: Vaccines (Basel). 2024 Jan 12;12(1):78. doi: 10.3390/vaccines12010078 (PMC10820992; doi:10.3390/vaccines12010078)
Supplement: Supplementary file 1 [file vaccines-12-00078-s001.zip › vaccines-2746797-supplementary.pdf]

a.

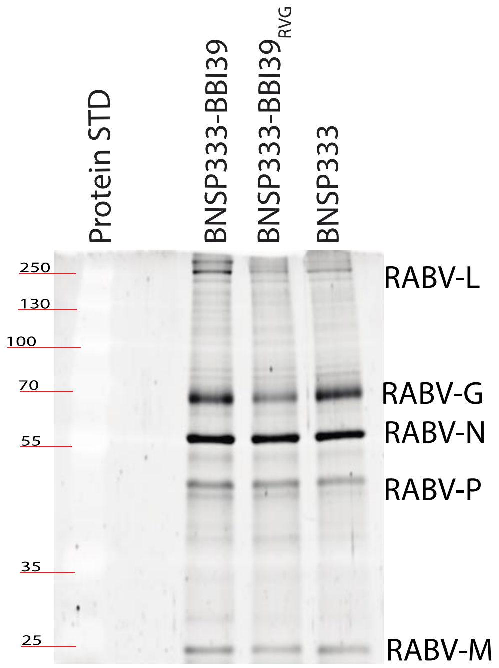

Supplementary Figure S1:

SDS-PAGE protein gel of RABV-based constructs.

(a) Protein gel of sucrose purified virions showing all RABV proteins.

a.

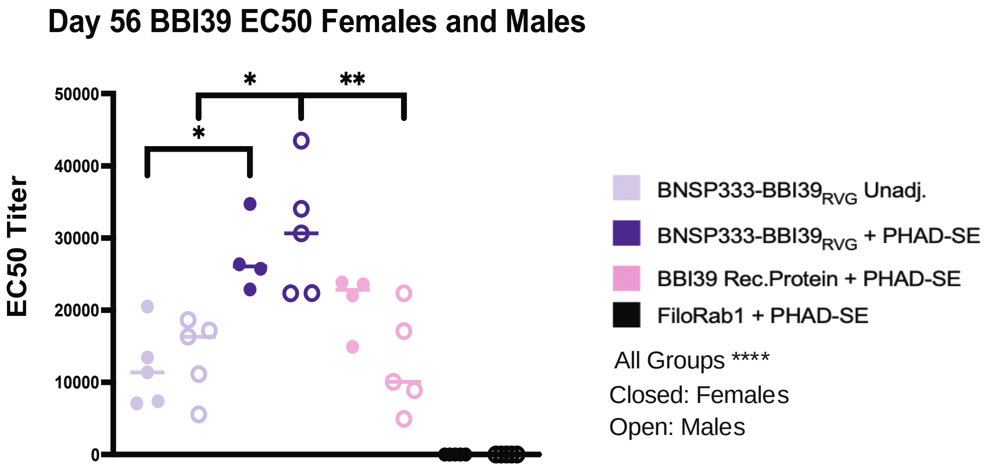

Supplementary Figure S2

Sex differences in antibody titers from vaccinated female and male mice

(a) anti-BBI39 total IgG EC50 titers for comparing male and female vaccinated mice. Statistics were calculated by one-way ANOVA with post hoc Tukey's test of log-transformed data. p > (ns), p < 0.0332 (*), p < 0.0021 (**), p < 0.0002 (***), p < 0.0001 (****).

a.

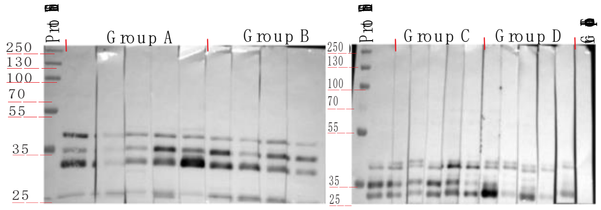

b.

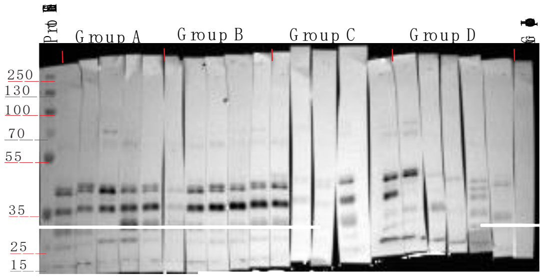

c.

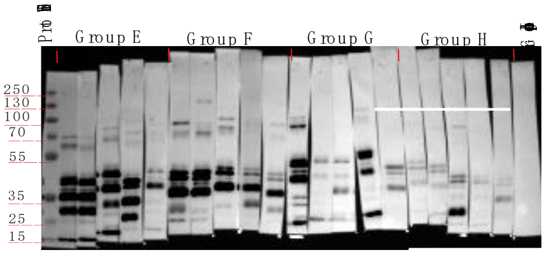

d.

	Groups	Number of Infected Mice		
		Heart	Joint	Skin
Group A/E	BNSP333-BBI39$_{RVG}$ Unadjuvanted	7/15	7/15	11/15
Group B/F	BNSP333-BBI39$_{RVG}$ + PHAD-SE	4/15	8/15	11/15
Group C/G	BBI39 Protein + PHAD-SE	9/15	10/15	12/15
Group D/H	FiloRab1 + PHAD-SE	15/15	15/15	15/15
Group E/I/J	Unvaccinated/Unchallenged	0/15	0/15	0/15

Supplementary Figure S3

Borrelia burgdorferi **depletion in infected organs by culture in BSK and confirmation of infection by western blot.**

(a-c) Western blot analysis of infected mice by serum. Whole borrelial lysates ran on gel and transferred to nitrocellulose membrane. Probed with individual infected mouse serum from 2 separate experiments of female (a-b) and male (c) mice. (d) Number of infected mouse organs by culture in BSK-H medium numerated under dark field microscopy for presence of *Borrelia*.

a.

	Groups	Number of Infected Mice: Heart	Joint	Skin
Group A	BNSP333-BBI39$_{RVG}$ Unadjuvanted	3/5	3/5	4/5
Group B	BNSP333-BBI39$_{RVG}$ + PHAD-SE	5/5	2/5	2/5
Group C	BBI39 Protein + PHAD-SE	4/5	1/5	4/5
Group D	FiloRab1 + PHAD-SE	5/5	5/5	5/5
Group E	Unvaccinated/Unchallenged	0/5	0/5	0/5

b.

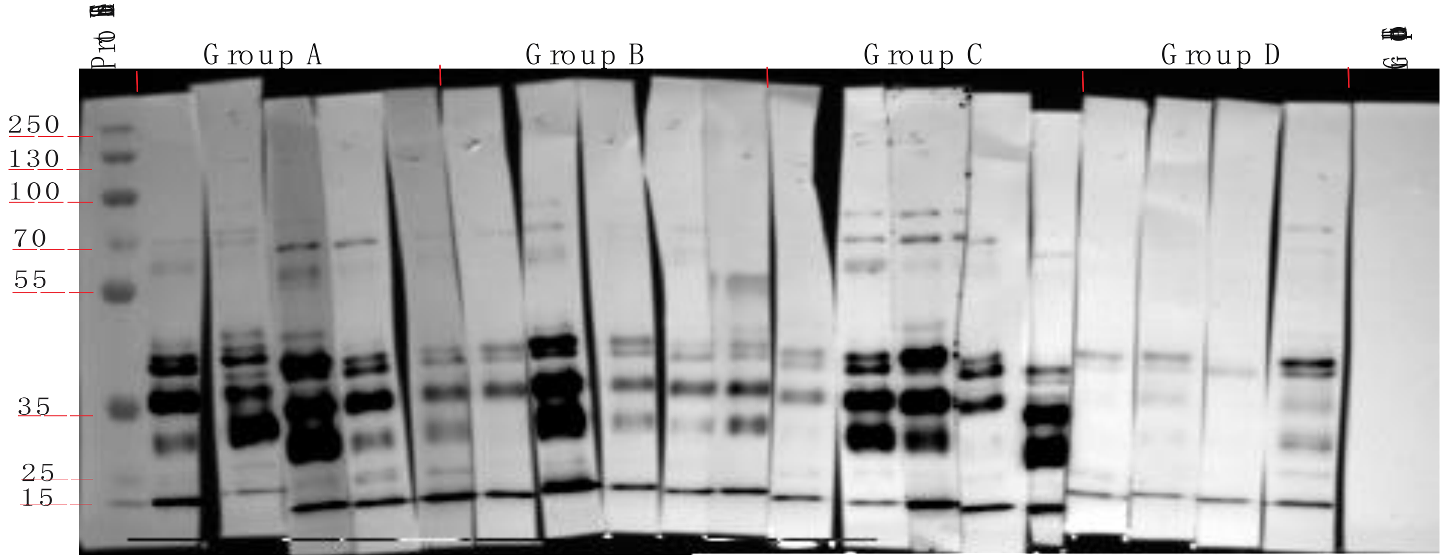

Supplementary Figure S4

Long-term vaccination and challenge organ culture and western blot analysis.

(a-b) ELISA curves for anti-BBI39 and anti-RABV-G from individual mouse serum 8 months (224 days) post-initial immunization. Determination of EC50 titers in Figure 7.

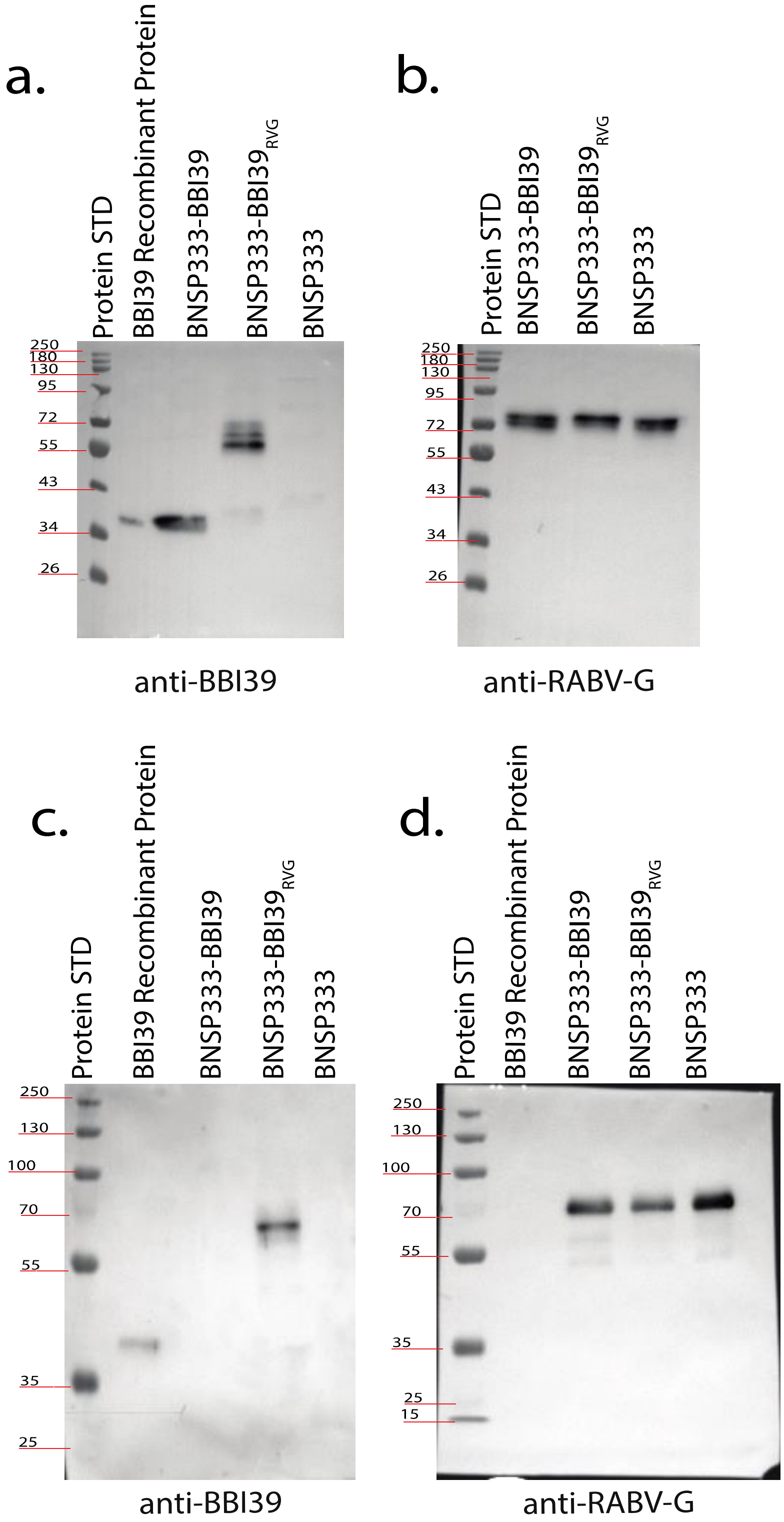

Supplementary Figure S5:

Raw data of western blots from Figure 2.

Western blot from Figure 2b (a-b) and Figure 2d (c-d).
